# p53 at the Crossroads between Different Types of HDAC Inhibitor-Mediated Cancer Cell Death

**DOI:** 10.3390/ijms20102415

**Published:** 2019-05-15

**Authors:** Maria Mrakovcic, Johannes Kleinheinz, Leopold F. Fröhlich

**Affiliations:** 1 Department of Cranio-Maxillofacial Surgery, University of Münster, Albert-Schweitzer-Campus 1, 48149 Münster, Germany; maria.mrakovcic@web.de (M.M.); johannes.kleinheinz@ukmuenster.de (J.K.); 2 Department of Medical Microbiology, University of Münster, Albert-Schweitzer-Campus 1, 48149 Münster, Germany

**Keywords:** HDAC, HDACi, SAHA, autophagy, p53, apoptosis, tumor, cancer, cell death

## Abstract

Cancer is a complex genetic and epigenetic-based disease that has developed an armada of mechanisms to escape cell death. The deregulation of apoptosis and autophagy, which are basic processes essential for normal cellular activity, are commonly encountered during the development of human tumors. In order to assist the cancer cell in defeating the imbalance between cell growth and cell death, histone deacetylase inhibitors (HDACi) have been employed to reverse epigenetically deregulated gene expression caused by aberrant post-translational protein modifications. These interfere with histone acetyltransferase- and deacetylase-mediated acetylation of both histone and non-histone proteins, and thereby exert a wide array of HDACi-stimulated cytotoxic effects. Key determinants of HDACi lethality that interfere with cellular growth in a multitude of tumor cells are apoptosis and autophagy, which are either mutually exclusive or activated in combination. Here, we compile known molecular signals and pathways involved in the HDACi-triggered induction of apoptosis and autophagy. Currently, the factors that determine the mode of HDACi-elicited cell death are mostly unclear. Correspondingly, we also summarized as yet established intertwined mechanisms, in particular with respect to the oncogenic tumor suppressor protein p53, that drive the interplay between apoptosis and autophagy in response to HDACi. In this context, we also note the significance to determine the presence of functional p53 protein levels in the cancer cell. The confirmation of the context-dependent function of autophagy will pave the way to improve the benefit from HDACi-mediated cancer treatment.

## 1. Introduction: Cell Death and Cancer

Resistance to programmed cell death and uncontrolled cell proliferation are pivotal landmarks supporting the initiation and progression of the complex and widespread disease known as cancer. The concept of programmed cell death as a multi-step, non-accidental, tightly controlled and defined self-destruction process was uncovered in 1964 [1]. The main forms of programmed cell death that were initially identified based on morphology are apoptosis, autophagy, and necroptosis; which, following their disruption, facilitate the development and growth of cancer [2,3,4,5]. An imbalance between cell division and cell death is a crucial Janus-faced factor, not only in tissue homeostasis and development, but also in the initiation, progression, and treatment of cancer, as it can be the cause as well as the solution to the dilemma [6]. Comparable to other defined common traits that govern oncobiology, such as limitless replicative potential, insensitivity to anti-growth signals, sustainment of angiogenesis, the ability to form metastases, deregulated metabolism, and the escape from the immune system, the inactivation of cell death mechanisms by cancer cells has been related to genetic defects in oncogenes or tumor suppressor genes [7,8]. In recent years, tumor initiation and progression has been increasingly linked to the disruption of epigenetic modifications, including DNA methylation, posttranslational histone or non-histone modifications, and microRNAs which may be considered as a further distinguishing mark of cancer [9]. In fact, several studies have confirmed that epimutations concerning the structure of chromatin are mutational hotspots in cancer representing about one third of so-called tumor-causing driver mutations [10,11]. Consequently, these aberrant epigenetic determinants have also been identified in the deregulation of different key signaling pathways of the cell, including those for cell death mechanisms. Targets for these epigenetic changes include, oncogenes and tumor suppressor genes, other key regulatory molecules, or even microRNAs that regulate apoptosis and autophagy.

The tumor suppressor and non-histone protein p53 is allocated an important role in this context. The modification of the acetylation of p53 regulates its transcriptional activity, related among many other functions to the regulation of apoptosis and autophagy, which is pivotal for the elimination of malignant cells [12]. The present review aims to address the ambiguous role of p53 as a modulator of the nature of histone deacetylase inhibitor (HDACi)-mediated cell death response in tumor cells, in particular for the induction of autophagy in cancer therapy. In view of our recent finding, we highlight the supposed function of p53 as a molecular switch that directs HDACi-mediated tumor response towards either forms of programmed cell death depending on its presence in the cell [13,14,15].

## 2. Post-Translational Acetylation Defects in Cancer

Histones are the core proteins that enable the higher organization of eukaryotic genomic DNA into nucleosomes [16]. Among the increasing number of possible post-translational histone modifications, the acetylation of histones, as well as non-histone proteins, have been recognized as a major epigenetic regulatory mechanism of gene transcription [17]. Thereby, the chromatin structure and regulation of gene expression of numerous key cellular processes, such as cell proliferation, differentiation, adhesion, and cell death is strongly affected [18,19]. Conclusively, histone acetylation has become apparent as a mechanism that enables the cell to deal with a variety of stressful conditions. Histone and non-histone protein acetylation is accomplished by different enzyme classes of histone acetyltransferases (HAT) and histone deacetylases (HDAC) that regulate the structure of chromatin and thereby modulate the accessibility of transcription factors to their target DNA sequences [20]. They belong to the so-called bromodomain proteins which are able to identify acetylated lysine residues on the N-terminal tails of histones and translate this signal [21]. Histone acetylation, particularly as proximal promoters and enhancers of transcriptionally active regions, untighten the connection between core nucleosome proteins and DNA, and thus facilitate the binding of transcription factors. One of the first recognized and decisive non-histone proteins targeted by HDACs and HATs, is the transcription factor p53 that regulates cell apoptosis and autophagy among its multitude of other functions as the “gatekeeper” of the cell [22].

Aberrant histone and non-histone deacetylation were also identified as the cause of transcriptional repression of tumor-related genes that interfere with important physiological cellular processes such as cell death regulation, and thereby promote malignant transformation [23,24]. Dysregulation, caused by the overexpression, abnormal HDAC recruitment, or loss-of-function mutations of HDACs in human cancer have been detected in solid (neuroblastoma, medulloblastoma, lung, gastric, liver, pancreatic, colorectal, breast, ovarian, prostate, renal, bladder, melanoma, oral, endometrial, pancreatic, thyroid, esophageal) as well as hematological tumors (ALL, CLL, AML, DLBCL, CTCL, HL, Myeloma) [25,26,27]. In particular, the loss of acetylation of histone H4 at lysine 16 (H4K16ac) was found to be decisive in tumorigenesis [28,29]. This finding is underlined by the fact that global histone modifications, such as acetylation and dimethylation, or even trimethylation levels of histone H3 and H4 (H3K9ac, K18ac, H4 K12ac; H3 K4diMe, H4R3diMe; H4K20triMe) correlate with the risk of cancer recurrence and allow a prognosis of poor survival in distinct types of cancer [30,31]. In addition, also HATs have been shown to be involved in cancer among other malignancies caused by the altered acetylation of histone proteins and non-histone proteins [26,32]. However, their effects could be either tumor-promoting or tumor-suppressive due to the hypo- or hyperacetylation effects of oncogenes, respectively. Hyperacetylation via HAT p300, for example, was correlated with the occurrence of hepatocellular carcinoma and the specific acetylation of H3K18 with prostate cancer [33,34]. Furthermore, p300 as an important trascriptional co-activator of androgen receptor activity, was shown to be upregulated during prostate or colorectal cancer progression [35,36,37,38]. HATs have also been reported to acetylate non-histone targets, such as the oncoprotein c-Myc promoting increased stability and cancer progression, or the tumor suppressor protein p53, promoting its transcriptional activity and cytoprotective function [39,40,41].

## 3. HATs and HDACs, the Effectors of Post-Translational Acetylation and Deacetylation

The dynamic and reversible acetylation of proteins is catalyzed by the post-translational actions of the families of HAT and HDAC enzymes which represent lysine acetyltransferases or deacetyltransferases, respectively [42]. Acetylation belongs to a myriad of possible histone modifications such as methylation (mono-, di-, and tri-methylation), phosphorylation, ubiquitination, sumoylation, or unfamiliar variants, such as succinylation, butyrylation, and neddylation, that have been intensively investigated because of their fundamental role in regulating gene expression by altered chromatin structure [19,43,44,45,46,47]. While HATs assist in the transfer of an acetyl group from acetyl-CoA to form ε-*N*-acetyllysine residues of histone and non-histone proteins and activate gene transcription by forming an open chromatin configuration associated with euchromatin, HDACs remove it, and consequently suppress transcription by supporting a condensed configuration [17,48,49]. Particularly, DNA elements that have regulatory function, such as promoters, or the histones H3 and H4, that are part of the tetrameric nucleosome structure and regulate transcriptional access, undergo specific acetylation [19,50]. In addition to transcriptional regulation, many essential cellular basic activities are governed by specific HAT/HDAC activity, which is primarily due to the vast number of non-histone proteins that have been identified as their targets, and seem to be their evolutionary primary targets [18,51]. These include, in addition to the regulation of cell death, replication, DNA repair, cell cycle regulation, DNA stress response, and senescence among others. Via modifying the acetylation of non-histone proteins, many molecular functions can be influenced, such as mRNA splicing, mRNA transport and integrity, protein translation, protein activity, localization, stability and interactions [18]. As non-histone substrates, tumor suppressor proteins (e.g., p53, RUNX3), signaling mediators (e.g., STAT3, β-catenin, SMAD7), steroid receptors (e.g., androgen, estrogen, SHP), transcriptional factors, and co-regulators (e.g., c-MYC, HMG, YY1, EKLF, E2F1, GATA factors, HIF-1α, MyoD, NF-κB, FoxB3), as well as structural (e.g., cell motility proteins), chaperone proteins, and nuclear import proteins (e.g., α-tubulin, importin-α, Ku70, HSP90) have been identified [52,53,54].

Depending on their subcellular localization, in either the nucleus or cytoplasm, HATs are categorized into Type A or B, that either contain or lack a bromodomain, respectively [55,56]. Based on sequence homology as well as shared structural features and functional roles, they have been grouped into the three major categories of GNAT (GCN5-related *N*-acetyltransferases), EP300/CREBBP (E1A binding protein p300 and CREB-binding protein), and MYST family [48]. PCAF (p300/CBP-associated factor), belonging to the GNAT family, and the paralogues p300 and CBP, which are key transcriptional co-activators of the cell and the only members of the EP300/CREBBP family, are the most important non-histone acetylating HATs. Although HATs are implicated in tumor progression, and in vitro designed inhibitors might be potentially useful, these are not clinically applicable thus far. Due to their function in protein complexes, current HAT inhibitors experience disadvantageous properties such as instability, low potency, or a lack of selectivity [57]; nevertheless, although progress on the advancement of clinically useful HAT inhibitors is delayed, they will be of interest for future cancer therapy [58].

According to their structure and homology to yeast proteins, the 18 known HDACs of humans have been divided into four classes. These comprise class I HDACs (with members HDAC1, 2, 3, 8), class II HDACs (with members HDAC4, 5, 6, 7, 9, 10), class III HDACs which were termed sirtuins (with members SIRT 1-7), and class IV HDACs (with member HDAC11). Only class III HDACs, containing the sirtuins, require NAD+ for their activity while all other classes contain a zinc-binding site. Also, differing subcellular localization can be applied for HDAC classification [49]. In contrast to the more restricted tissue-specific expression pattern of the HDAC classes II to IV with subcellular localization in nucleus, cytoplasm, or mitochondria, class I exhibits a ubiquitous distribution pattern due to the exclusive presence in the cell nucleus explaining the high enzymatic activity of its members. Thus, for example, sirtuins have been found to reside in all three compartments including SIRT1 and 2 in the cytoplasm, SIRT3, 4, and 5 in mitochondria, and SIRT1, 2, 6, and 7 in the nucleus. The subcellular presence of HDAC6 and 10 mostly in the cytoplasm is another reason for the separation of these members of HDAC class II A from B, as they can be localized in the nucleus, cytoplasm (HDAC4, 5, 9), as well as in mitochondria (HDAC7).

## 4. Inhibition of Post-Translational Deacetylation in Cancer Cells

Related to these findings of post-translational acetylation defects in cancer, increasing interest has been attracted by the development of HDAC inhibitors (HDACi) as encouraging candidates for cancer therapy that are able to reverse the actions of deregulated HDAC expression [59,60]. Comparable to other epigenetic drugs, such as to DNA methyltransferase (DNMT) inhibitors, HDACi are able to lift the silenced expression of tumor suppressor genes and reactivate associated signaling pathways such as programmed cell death. As opposed to unalterable genetic mutations, these compounds that modify acetylation or methylation attachments of protein and DNA molecules make use of the partial reversibility of the epigenome. Although several drawbacks for solid tumors exist, many in vitro data and clinical studies have already attested positive effects for the use of HDAC targeting; however, their detailed mechanisms of action are still are under investigation. Moreover, further inhibitors are in the process of development. Nevertheless, the prediction of the cellular or molecular response following HDACi treatment remains elusive as they may exert pleiotropic effects that vary, depending on the class of HDACi and the type of tumor. Therefore, a clear-cut strategy or definition of predictors for the use of HDACi in cancer therapy would be required. Improved molecular mechanistic insights related to cell death and resistance will therefore be useful for enabling a more targeted use of HDACi and the prediction of beneficiaries in cancer therapy [61].

HDACi categories encompass hydroxamic acids (hydroxamates), cyclic tetrapeptides, benzamides, electrophilic ketones, and aliphatic acids that include natural but also synthetic derivatives [62]. This classification mainly relies on the chemical structure of their zinc-binding group; in addition, HDACi can also be subdivided into zinc-dependent pan- or broad-spectrum inhibitors that inhibit class I, II and IV HDACs in contrast to primarily class I-specific HDACi [23,63,64]. Favored representatives of the hydroxamates are SAHA (suberoylanilide hydroxamic acid, vorinostat, Zolinza), which is a preferred derivative of naturally occurring TSA (trichostatin A), as well as the CBHA (m-carboxycinnamic acid bishydroxamate)-derived tubacin, LAQ-824 (dacinostat), LBH-589 (panobinostat), or PXD-101 (belinostat) [64,65,66,67]. The class I-selective FK-228 (romidepsin, FR901228, istodax) belongs to the group of cyclic tetrapeptides [68,69,70]. MS-275 (entinostat) and MGCD0103 (mocetinostat), exhibiting enhanced HDAC class I selectivity, are members of benzamide-based HDACi [71,72,73]. The minor effective class I- and IIa-specific HDACi, VPA (valproic acid), PBA (phenylbutyrate), NaB (sodium butyrate), or AN-9 (pivaloyloxymethyl butyrate) belong to the category of aliphatic acids [74,75].

The unexpected, tumor-specific effects of HDACi in contrast to the ubiquitous functions of HDACs in gene regulation remain puzzling; however, they commonly induce chromatin relaxation and transcriptional de-repression in cancer cells. By the reactivation of a small percentage of silenced key regulatory genes, e.g., tumor suppressor or oncoproteins, due to the restored or increased acetylation, re-expression of genes associated with multiple detrimental tumor-defensive mechanisms, such as cell cycle arrest, differentiation, and cell death, but also the inhibition of metastasis and angiogenesis, is implemented [76,77,78,79,80,81]. Accordingly, major efforts have been undertaken to employ HDACi for the development of promising novel anticancer strategies which have ended in the establishment of numerous clinical trials that involve HDACi (www.clinicaltrials.gov). Either monotherapeutic or combinatorial treatments of several HDACi in hematological and solid malignancies with varying outcomes, have been, or are in the process of being tested [82,83,84]. Up to now, these evaluations have led to the admittance of four licensed HDACi (SAHA, panobinostat (LBH589), belinostat (PXD-101), and romidepsin (FK228)) for the treatment of cutaneous T cell lymphoma, multiple myeloma, or peripheral T cell lymphoma representing exclusively pan-inhibitors [85,86,87,88,89]. Although preclinical studies involving single treatment regimen of many HDACi were promising, almost all types of solid tumor (e.g., ovarian, breast, renal, prostate, and head and neck cancer) failed to demonstrate benefits in phase II clinical trials [90,91,92,93,94,95,96]. Moreover, patients experienced considerable non-selective off-target effects ranging from negligible (such as diarrhea, anorexia, dehydration) to serious (myelosuppression, thrombocytopenia, and cardiotoxicity) outcomes [75,84,97,98]. The reasons therefore are currently unclear and were discussed to be due to a combination of divergent blood vessel supply, intrinsic molecular heterogeneity involving epigenetic modifications, and the progression of treatment resistance. Consequently, nanoparticle-supported targeting has been proposed as a future drug-delivery method to avoid potential stability issues with HDACi drugs; however, also in this case negative long-term treatment effects might be predicted [99,100]. Therefore, to bypass clinical limitations and reduce adverse effects, the development of isoform-specific and/or tissue-specific HDACi with improved selective efficacy, in place of pan-inhibitors, is enforced [63,101]. Collectively, the improved understanding of the context-dependent effects in epigenetic interference mediated by individual HDAC enzymes will provide a synergistic advantage for designing specifically targeted cancer treatment strategies.

## 5. Effector Mechanisms of HDACi-Induced Cell Death

Due to a range of histone and non-histone targets and multitude of effects exerted by the posttranslational modification of acetylation, HDACi can arrange an array of cellular effects with respect to growth, differentiation, migration, senescence, and death that contribute to their eminent anti-tumor activities [102]. These may differ, depending on the type of tumor and applied dose; nevertheless, specific mechanisms are crystallizing for many HDACi [98,102,103,104]. It is very likely that future research will shed light on additional, non-elucidated mechanisms contributing to HDACi-mediated anticancer activity.

As a prerequisite for the induction of programmed cell death, a fundamental effect of HDACi was determined by their ability to re-induce cell cycle arrest and induce cell differentiation in transformed cells by downregulating positive regulators of cell proliferation, such as c-MYC and c-SRC [65,105,106,107,108,109,110,111]. Cell cycle arrest, in the G1 or G2 phase of the cell, is induced by p53-dependent or -independent induction of p21 (encoded by the gene *CDKN1A*) expression [112,113,114,115]. p21 is a cyclin-dependent kinase inhibitor that interferes with cyclin D1 and D2 activity [116,117]. Presumably, due to accumulated DNA damage (e.g., by DNA double-strand breaks caused by oxidative stress), this failure to exit the cell cycle from an incomplete mitosis might result in the induction of apoptosis [118,119,120,121]. Consistently, upon inhibiting the SIRT1-mediated deacetylation activity of p53, DNA damage-induced apoptosis was activated by p53-mediated target gene expression in a representative report [122]. The induction of DNA damage and cell death goes along with another very important characteristic mechanism of HDACi, which is the generation of reactive oxygen species (ROS). Accordingly, the accumulation of thioredoxin (TXN), a natural scavenger of ROS and intracellular antioxidant, was detected in normal, but not in malignant, human fibroblasts [123]. Interference with multiple DNA repair processes in response to various genotoxic insults, or the induction of autophagy, is a novel discovered effector mechanism important for the maintenance of genomic integrity. The inhibition of HDAC6, for example, was documented to provoke cell death as the MSH2-regulated DNA mismatch repair capability of the cell was deregulated [124]. This is a very obvious finding, as HDACs have been reported to modulate deacetylation of histones at DNA damage sites, and are fundamentally involved in regulating the expression of DNA damage-related response proteins [25,125,126,127,128,129,130,131,132,133]. In support of this, increased expression of a marker of DNA double strand breaks, H2AX, was disclosed in transformed but not in normal cells in response to SAHA treatment, explaining its tumor cell-specific activity [120]. Furthermore, by directly altering the acetylation pattern of non-histone proteins such as transcription factors, the signaling pathways that are involved in cell death (e.g., NF-κB, p53, and STATs), can be re-activated by HDACi in cancer cells [18]. For instance, the half-life of the transcriptional activity of p53 is determined by its acetylation status which regulates its degradation through the directly interacting MDM2 E3-ligase in H1299 carcinoma cells [134]. Further detrimental effects, resulting from HDACi treatment, are the interference with chaperone protein function and the inhibition of stress response pathways in the endoplasmic reticulum [107,135]. Thereby, the physiological elimination of misfolded proteins, as well as the stability and expression of oncoproteins, can be affected. Moreover, HDACi were found to interfere with the migration and invasion of human tumor cell lines by de-repression of metastasis-related genes [136,137]. Additionally, the inhibition of angiogenesis by changing the expression of many pro- and anti-angiogenic genes was attested for HDACi [138,139,140].

Overall, key determinants of HDACi lethality that interfere with cellular growth in a multitude of tumor cells, are apoptosis and autophagy [141,142,143,144]. These morphologically distinct two major forms of programmed cell death which promoted the use of HDACi as promising therapeutic agents will be discussed in the next two sections.

### 5.1. HDAC Inhibitor-Induced Apoptosis

For many years, apoptosis has been considered as the prevailing mechanism of programmed cell death in eukaryotic cells which is traditionally targeted in cancer therapy. Also, cells undergoing HDACi treatment exhibit caspase-mediated induction of apoptosis, as the most commonly encountered form of cytotoxic response [145,146,147]. Two different modes—either the intrinsic (mitochondrial) pathway, or the extrinsic (death-receptor) pathway—can be employed, alone or in combination, which finally both discharge executioner caspases that degrade and eliminate the cell [145,148]. As already mentioned above, this programmed type-I cell death is activated by HDACi exclusively in cancer cells, presumably by rendering them sensitive due to the imposition of cell cycle arrest [149].

**Figure 1:**
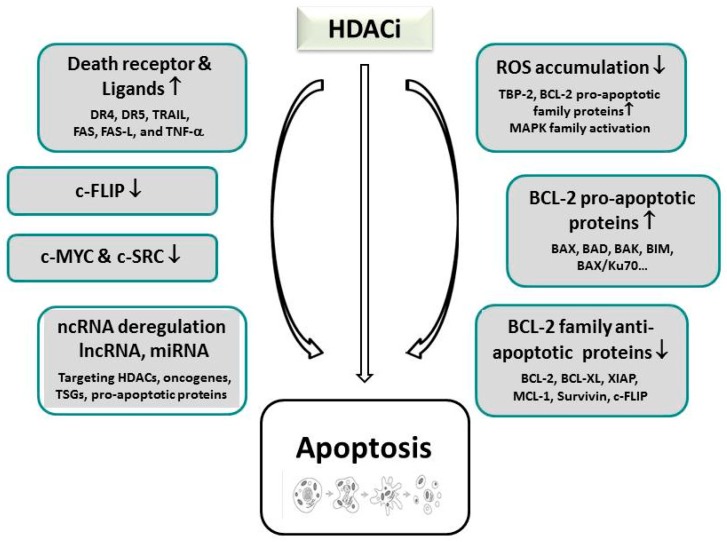
Known mechanisms of histone deacetylase inhibitor-induced (HDACi) re-activation of apoptosis. These activate the intrinsic (mitochondrial) the extrinsic (death-receptor) or both pathways of apoptosis. Also non-coding RNAs, of which microRNAs prevail, have been described to mediate HDACi-stimulated apoptosis.

Upregulation of pro-apoptotic proteins belonging to the B-cell Lymphoma-2 (BCL-2) family (e.g., BAX and BAK) that comprise the modifying members of the BH3 (BCL-2 homology 3)-only protein family, including sensitizers (NOXA, BAD) and activators (BID, BIM, BMF, PUMA), lead to the disruption of the mitochondrial membrane, the release of cytochrome C, apoptosome formation, and the final activation of executioner caspases in the intrinsic pathway of apoptosis [112,119,150,151,152,153]. Mechanistically, to elicit apoptosis in the intrinsic pathway, HDACi predominantly seem to interfere with the balanced expression between pro- and anti-apoptotic proteins, whereas a single case documented SAHA-induced cleavage of BID (see Figure 1) [119,149,154]. In addition to p53-dependent or -independent pathways, that will be discussed in Section 6.2, HDACi treatment can additionally activate ROS-dependent apoptosis by upregulation of pro-apoptotic proteins, and the induction of DNA damage [107,119,123,155,156]. As an underlying mechanism, elevated expression of the thioredoxin (TRX)-binding protein-2 (TBP-2) are assumed that are present in malignant cells; these inhibit the ROS scavenger TRX, which is able to compensate oxidative stress, [107,123]. For the enhancement of apoptosis, HDACi can also promote the down-regulation of anti-apoptotic genes (e.g., *BCL-2*, *BCL-XL*, *XIAP*, *MCL-1*, *Survivin*) [112,113,157,158]. As a consequence, HDACi-mediated apoptosis can be suppressed by the overexpression of BCL-2 and BCL-XL [119,145]. Similarly, interference of p53 with the interaction of BAX and Ku70, an acetylation-sensitive DNA repair protein, was shown to promote mitochondria-mediated apoptosis in neuroblastoma cells [159]; the involvement of p53 could be demonstrated by transactivation-deficient p53 mutants that were still able to mediate BAX-dependent apoptosis in HDACi-treated p53-null cells due to the transcription-independent activation of BAX.

In the extrinsic pathway, HDACi primarily restore the expression of death receptors such as DR4 and DR5, or their corresponding ligands, such as TRAIL, FAS, FAS-L, and TNF-α [160,161,162,163,164,165,166,167]; this reactivation very often triggers a bottleneck of apoptosis resistance along this pathway in untreated tumor cells and link the death receptor with the intrinsic pathway [145,168]. The interaction of these members of the TNF-superfamily then further support the recruitment of the death-inducing signaling complex (DISC), which contains the FADD adapter protein and the initiator caspases-8 and -10 [169,170]. Also for the death receptor pathway, HDACi-stimulated expression of anti-apoptotic proteins such as of cytoplasmic FLICE-like inhibitory protein (c-FLIP) were noticed [171].

Recently, also non-coding RNA (ncRNA) expression, mostly in the form of microRNAs, has been implicated in HDACi-reactivated induction of apoptosis in cancer cells. Importantly, these have been demonstrated to take over the functions of tumor suppressor proteins and underlie epigenetic regulation themselves; accordingly, microRNAs such as microRNA (miR)-127, miR-124a, and miR-34b/c, have been methylated in cancer cells, thus generating many potential targets of cancer therapy [172]. Apoptosis was mediated in these reports either by deregulating miRs that target HDACs, thereby mimicking HDACi-inducible effects, or by directly interfering with the expression of miRs. The first mechanism was documented for instance for HDAC1 that was repressed by miR-449a in prostate cancer [173]. Examples for direct regulation of miR-supported apoptosis were documented by TSA and SAHA-induced overexpression of miR-129-5p in thyroid cancer cells, or c-MYC-mediated silencing of miR-15 and let-7 miRs that cause transcriptional suppression of BCL-2 and BCL-XL in B-cell lymphomas [174,175]; furthermore, sodium butyrate and panobinostat-induced expression of miR-31 caused apoptosis and cellular senescence via downregulation of BIM1 in breast cancer cells and fibroblasts [176]. A sophisticated role could be unraveled for miR-34a in pancreatic cancer by engaging as a tumor suppressor miR and even regulating acetylation in a feedback loop [177]. SAHA treatment re-established miR-34a expression that led to p53 acetylation and consequent upregulation of *CDKN1A* as well as *PUMA* (p53 upregulated modulator of apoptosis) target genes, thereby initiating the induction of apoptosis.

Direct assessment of the role of HDACs has also yielded several candidates that were implicated in the regulation of intrinsic apoptosis by interfering with the subtle balance of pro-apoptotic and anti-apoptotic factors. The deletion of HDAC2 in gastric cancer cells promoted the upregulation of the proapoptotic proteins BAX, AIF, and APAF-1, while it silenced the expression of BCL-2 [178]. The BCL-2 modifying factor, BMF which is a pro-apoptotic activator, was reported to be conjointly downregulated by HDAC1 and 8; inhibition of HDAC8 by methylselenopyruvate in colon cancer cells restored BMF downregulation and thereby activated apoptosis [150,179]. HDAC3 was found to suppress the pro-apoptotic protein PUMA in gastric cancer cells which can be restored by HDACi (TSA) treatment [180].

### 5.2. HDAC Inhibitor-Induced Autophagy

Autophagy is a conserved catabolic cellular mechanism of self-degradation of cytoplasmic constituents. Autophagy has been categorized into macroautophagy, microautophagy, and chaperone-mediated autophagy of which we further discuss macroautophagy in here, if not stated otherwise [181,182,183,184]. Known signals triggering autophagy are quite diverse and include mostly shortage of nutrients but also the presence of protein aggregates, damaged organelles, hypoxia, and ROS. Aged or damaged molecules and organelles are recycled in specifically for this process formed autophagosomes which is governed by a complex genetic program required in cellular homeostasis or cell death [185,186]. Unlike apoptosis or necrosis, autophagy has been attributed with a dual role in cancer which can result either in a survival- or a death-promoting response to encounter adverse genotoxic or pharmacological stress. This form of HDACi-incurred lethality has only recently been brought into evidence as an effector mechanism that interferes with cellular growth [187]. Epigenetic interference in the regulation of autophagy can either inhibit, or support, the formation of a malignant phenotype. The complex cytoprotective or cytotoxic response of autophagy in tumor cells thereby seems to depend on the type and stage of cancer, its genetic predisposition, as well as the duration and dose of HDACi treatment [188,189,190,191]. The cellular response might also reflect the diverse mutational status of cancer cells, in particular with regard to the highly altered oncoproteins or oncosuppressor genes, such as *AKT-1*, *PTEN*, *BECLIN-1*, or *p53* that promote tumorigenesis and are crucial regulators of autophagy [192]. A further issue, why this type of mostly pathological or drug-induced cell death is controversially discussed, might be found in the largely unknown mechanisms that determine how autophagy eliminates cells. One explanation could be the selective accumulation and degradation of cell survival factors in autophagosomes; thus, accumulation of ROS and cell death could be induced by the recruitment of catalase in autophagososmes [193,194]. In general, it has been elaborated that autophagy prevailingly exerts a protective and tumor-suppressive role during the initial phases of tumor development, but also in normal cells. This kind of surveillance mechanism helps to reduce the effects of ROS accumulation by removing damaged organelles and cellular components, and by decelerating the transformation potential of healthy towards malignant cells [195]. For instance, it was evident in mice with a hemizygous Beclin-1 deletion that predisposed for increased tumor formation, or in autophagy-mediated clearance of SAHA-treated apoptosis-resistant uterine sarcoma cells [196]. The tumor-promoting effects of autophagy, however, seem to outweigh in the later progression and metastasis stages of established tumors. Here, autophagy eradicates ROS-induced metabolic stress products, provides nutrients required for cancer cell survival, and mediates resistance during chemotherapy to prevent tumor cell elimination [197]. Under these pathological stressful conditions, autophagy can be stimulated above the usual basal levels that are present in normal cells, and cell death is presumably favored by enhancing the “self-eating” program by not yet elucidated mechanisms [198]. The disruption of autophagy, either by endogenous defects, or by pharmacological interference, as for instance by HDACi, will therefore lead to prolonged tumor survival as it potentiates cell death. This insight is particularly relevant, since HDACi-mediated inhibition of autophagy in combination with chemotherapeutic treatment has been favored as a novel approach in cancer therapy. Consequently, the confirmation of the context-dependent function of autophagy and investigation of limiting molecular determinants gain tremendous importance for a positive therapeutic outcome [191,199,200].

**Figure 2:**
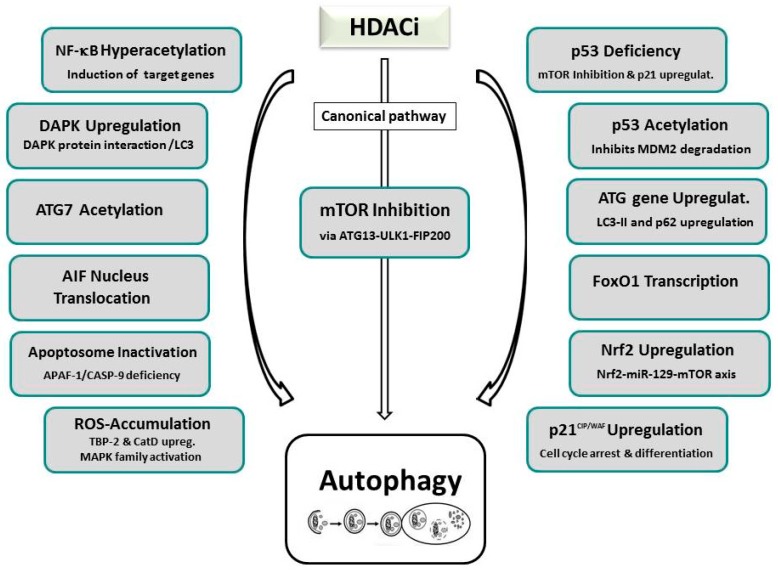
Known mechanisms and signaling pathways involved in histone deacetylase inhibitor-activated induction or suppression of autophagy. In most cases mTOR inhibition representing the canonical pathway, ROS accumulation, NF-κB hyperacetylation, p21 upregulation, or the involvement of p53 signaling is observed.

Research thus far has unveiled a multitude of HDACi-induced suppressive or activating autophagic signaling pathways for autophagy involving primarily the modification of HAT/HDAC-mediated acetylation of many “AuTophaGy-related” (ATG) proteins, including ULK1 (UNC-51-like kinase 1), ATG3, or ATG7 (see Figure 2) [14,98,201]. A major frequented pathway of HDACi-induced autophagy is represented by suppression of mTOR, the nutrient-sensing kinase mammalian target of rapamycin, that is often accompanied by increased expression of ATG proteins or regulators, including LC3 (microtubule-associated protein 1A/1B-light chain 3) and BECLIN-1 [13,187,202,203,204,205,206,207,208]. mTOR is a known major molecular hub of autophagy, that activates autophagosome formation by inactivating another major downstream autophagic switch, the ULK1 complex. The fundamental role of mTOR by SAHA-induced inactivation leading to re-establishment of ULK1 function could initially be documented by studies of our own group and by Gammoh et al. on endometrial sarcoma cells, and were confirmed in several subsequent cases [13,202,203,204,205,206]. Many research groups also published an additional occurrence of apoptosis in cells that underwent HDACi-induced autophagy. Alone or in combination with mTOR deactivation, massive intracellular ROS generation impairing mitochondrial respiration and energy metabolism has been reported to mediate SAHA-facilitated autophagy in tumor cells. In several tumors undergoing HDACi-dependent ROS accumulation, additional increased expression of the lysosomal protease cathepsin D, decreased expression of its substrate, TRX, or activation of ERK1/2 and JNK, that belong to the MAPK (mitogen activated protein kinase) family, has been resolved [203,209]. Mechanistically, enzymes related to energy metabolism, anti-oxidative stress, and cellular redox control are presumed to be involved in ROS-activated autophagy as evidenced by proteomic analysis of SAHA-induced Jurkat T-leukemia cells [203,210].

Either induction of NF-κB associated target genes involving hyperacetylation of NF-κB RELA/p65 in SAHA/MS-275 treated PC3 cells, or attenuation of pERK/NF-κB signaling, accompanied by increased transcriptional activity of *CDKN1A*, were also determined as the cause of HDACi-induced autophagy [211]. HDACi-stimulated p21 expression was documented in H40 and SAHA-treated PC-3M and HL-60 cells resulting in cell cycle arrest, cell differentiation, and autophagy [212]. Furthermore, treatment of prostate cancer cells with the novel HDACi, MRJF4, promoted p21-elicited autophagy [213]. Further single reports, supporting other regulatory mechanisms of HDACi-promoted autophagy, include nuclear translocation of the apoptosis inducing factor (AIF), apoptosome inactivation, transcriptional activity of FoxO1, and the upregulation of death-associated protein kinase (DAPK) [13,211,214,215,216,217]. A very recent study even implicated microRNA-mediated regulation of mTOR by the transcription factor Nrf2 (nuclear factor erythroid 2 like-2) as a mechanism of HDACi-induced autophagy [218]. In response to HDACi treatment, increased Nrf2 mRNA and protein levels accumulated that led to the increased transcription of miR-129-3p which in turn targeted mTOR.

Nevertheless, the HDACi-mediated suppression of autophagy could be verified in two reports. In the first case, treatment of myeloid-leukemic cells with valproic acid, SAHA, TSA, panobinostat, or JQ2, decreased the expression of ATG7, an autophagic regulatory protein required for fusion of peroxisomal and vacuolar membranes, and thereby downregulated the autophagic flux but promoted autophagy [219]. Moreover, the sirtuin inhibitor tenovin-6 was demonstrated to suppress autophagy in chronic lymphocytic leukemia (CLL) cells as evidenced by increased expression of autophagy-lysosomal pathway genes and the autophagic markers LC3-II and p62 [220]. HDACi-dependent autophagic induction entailing p53 acetylation and p53-deficiency, based on our own findings, will be discussed in Section 6.2.

Involvement of members of different HAT families in the epigenetic control of autophagy via acetylation of different ATG proteins and effectors has also been observed. Thus, ATG7 has been reported to co-localize and interact with the EP300 acetyltransferase [221]. Silencing of EP300 by RNAi promoted the induction of autophagy while starvation-induced autophagy, a process controlled by BAG6/BAT3 (BCL-2-associated athanogene 6)-mediated regulation of EP300, was suppressed upon overexpression of EP300 [221,222,223]. Furthermore, beside the EP300 family, also the MYST family of HATs was associated with the induction of autophagy. TIP60-dependent acetylation of ULK-1 via glycogen synthase kinase-3 (GSK3) serves as a fundamental cellular mechanism that is initiated during starvation-induced autophagy [224]. The interaction of ATG3 with ATG8, that is required for autophagic pathway signaling, was found to depend on Esa1-mediated acetylation [49]. Histone H4 (H4K16ac)-mediated activation of autophagy was induced by the downregulation of hMOF (KAT8/MYST1) [50]. Also, histone H3 acetylation seems to induce autophagy as it was demonstrated to limit the development of resistance due to chronic mTOR inhibition in renal cell carcinoma cells that were targeted by valproic acid [225].

In contrast to the stimulatory autophagy-triggering signals caused by the majority of HDACi, HDACs, that initially trigger deacetylation activity, have been established as inhibitors of autophagy [226]. Either depletion of HDAC1, or HDACi (FK228)-mediated inhibition of HDAC1 in HeLa cells, induces autophagy as evidenced by accumulation of the autophagosomal marker LC3-II [227,228,229]. Combined inhibition of HDAC1 and 2 by TSA triggered phenylephrine-induced autophagy in cardiomyocytes by entangling the autophagic regulators ATG5 or BECLIN-1 [227,228]. Skeletal muscle-specific deletion of both HDACs therefore resulted in perinatal lethality in a subset of mice as they lacked skeletal muscle homeostasis and autophagic flux [230]. HDAC6 was established initially as a microtubule-associated deacetylase that, as a compensatory degradation mechanism, activates autophagy when the ubiquitin-proteasome-system is blocked [231]. In mitophagy—the autophagic degradation of mitochondria—a similar mechanism involving HDAC6 and SQSTM1/p62 recruitment by parkin-mediated ubiquitination was observed which assists in mitochondrial clearance [232]. The key role of HDAC6, that is able to bind to polyubiquitinated proteins and can be inhibited by tubacin, is the mediation of autophagosome-lysosome fusion by controlling the acetylation of SIK2 (salt-inducible kinase 2) [64,233]. HDAC6 seems to be a primary object regulating the anti-cancer response of HDACi by deacetylation of non-nuclear proteins; it is involved in the clearance of toxic misfolded protein aggregates through aggresome-mediated autophagy whose deficiency impairs tumor growth [234,235]. The induction of apoptosis as well as autophagy, in combination with cell cycle arrest and diminished ERK activation, were activated by Apicidin-elicited suppression of HDAC7 in salivary mucoepidermoid carcinoma cells [236]. HDAC10, presumably by modifying the acetylation of Hsp70 protein families, adds to autophagy-mediated cell survival as its disabling, either by ablation, or by treatment with class II-B inhibitors bufexamac and tubastatin, blocked the autophagic flux and prevented efficient autophagosome-lysosome fusion; hence, neuroblastoma cells were rendered more sensitive to cytotoxic drugs [237].

Among class III HDACs, SIRT1, 2, 3, 5, and 6 have been implicated in autophagic signaling. SIRT1 activity directly deacetylates ATG 5, 7, and 8 which are required for induction of starvation-induced autophagy [238]. Furthermore, participation in oxidative stress-induced autophagy in embryonic stem cells and PLB (Plumbagin)-induced autophagy in prostate cancer cells, involving the mTOR pathway, has been attested [239,240]. SIRT2 drives acetylation of FoxO1, a transcription factor that is regulated by ATG7 interaction and stimulates autophagy [241]. The mitochondrial deacetylase SIRT3 activates cytoprotective mitophagy in breast cancer and other cells during various conditions, such as ROS induction, starvation, or proteotoxic stress [242,243,244]. SIRT5 has been established in the downregulation of ammonia-induced autophagy and mitophagy by controlling glutamine metabolism [245]. By engaging the IGF-AKT-mTOR pathway, or by ROS-induced activation of mTOR, SIRT6 stimulated autophagy in human bronchial epithelial cells or neuronal cells, respectively [246,247].

## 6. The Role of p53 in HDACi-Mediated Cancer Cell Death

### 6.1. Post-Translational Regulation of Wild-Type and Mutant p53 by Acetylation

The transcription factor and tumor suppressor protein p53, considered as a “custodian” of the cell, has a detrimental role in the regulation of cell integrity and homeostasis and thus in tumor defense. This protein, p53, coordinates cellular responses such as cell cycle arrest, apoptosis, senescence, metabolism, differentiation, angiogenesis, and even modulates autophagy. Among many other post-translational modifications, acetylation assists this master regulator to sense and integrate a variety of internal and external cellular stress signals, such as DNA damage and changes in DNA methylation, genotoxicity, hypoxia, oxidative stress, or oncogene activation [22,44]. In response, p53, as a central transcription factor that translocates to the nucleus by detaching from the E3 ubiquitin ligase MDM2 (mouse double minute 2 homolog), modulates multiple downstream target genes that regulate processes such as cell cycle progression and cell death [134,248,249]. In appropriate conditions, p53 induces apoptosis by transactivating, i.e., transcriptional activation of pro-apoptotic genes, or by direct interaction with anti-apoptotic proteins located in the mitochondrial membrane [250,251].

p53 is a well-investigated representative of crosstalk with respect to acetylation, and other post-translational modifications [22,44,252]. Currently, 13 lysine residues of p53, that are located mostly at the C-terminal end, have been reported to undergo acetylation [253,254]. Acetylation and deacetylation of p53 are managed by the CBP/p300 or the MYST family (TIP60, hMOF) of HATs and by the HDAC1/mSin3 complex or Sirt1, respectively [22,255,256,257,258,259]. HDAC2 that is overexpressed in numerous cancer types has also been linked to the regulation of deacetylation of C-terminal lysine side-chains in p53, and functions as a corepressor that is involved in regulating to the interaction with target genes [260,261]. Furthermore, in human hepatocellular carcinoma tissue p53 protein levels were found overexpressed through downregulation of MDM2-mediated p53 degradation by SIRT3 [262,263]. Disruption of acetylation by HDACi at distinct sites could either affect the sequence-specific DNA binding activity which transactivate target genes or modify nuclear export, co-activator recruitment, or stability properties of p53 [12,22,252,256,257,264]. Examples are the failure of p53 to activate *p21* (*p21waf-1/cip1*) transcription, the ability to induce cell growth arrest upon deleting a C-terminal acetylation site, or the interference with ubiquitin–proteasome-dependent degradation of p53 by MDM2 in a feedback loop [265,266]. Reciprocally, by the HDACi-mediated hyperacetylation of key residues of p53, enhanced stabilization, cell cycle arrest, and the expression of pro-apoptotic genes can be achieved [267,268]. This finding gains particular importance as variants of p53, provoking a loss of the sequence-specific DNA binding ability of transcriptional targets, are among the most common mutations encountered in human cancer [248,269]. Hyperstable mutant gain-of-function variants which elicit overexpression of chaperone or MDM2 short isoforms, and accumulate in the cell due to increased half-life, can even have a dominant-negative effect on the remaining wild-type allele (e.g., by blocking access to the promoter, or by assuming pro-oncogenic functions) [270,271,272,273,274,275]. Consistently, HDACi treatment exerts a destabilizing effect by enabling the cell to degrade mutant p53, either by reactivating, or by mimicking its transactivation capability, resulting in increased *p21* and *MDM-2* expression [276,277]. Accordingly, a study using selective HDACi, exposed a regulatory mechanism of mutant p53 transcription which presumably involves direct interaction of HDAC8 via HoxA5, as histone acetylation is not altered [278]. Alternatively, HDACi (SAHA)-induced inhibition of HDAC6 was uncovered to facilitate the degradation of mutant p53 by MDM2 and CHIP ligases due to its release from the chaperone HSP90 [279]. Moreover, HDACi-induced autophagy was suggested as a further mechanism in eliminating mutant p53 [280,281].

### 6.2. HDACi-Mediated Apoptosis and Autophagy by p53

As mentioned earlier, the induction of mitochondrial and death receptor-mediated apoptosis that is often accompanied by ROS generation and p21-induced cell cycle arrest, has been denoted as the most common form of HDACi-mediated cell death [145,147,148]. Nevertheless, the role of p53 in this context is controversial as by HDACi caused post-translational acetylation, the capability of p53 to transcriptionally transactivate several pro-apoptotic genes, such as BAX, PUMA, and NOXA, is hindered. Thus, many studies demonstrate p53-independent p21 induction and apoptosis upon HDACi administration and the anticancer effect of HDACi is not influenced by the mutational status of p53 in the tumor [104,105,106,217,282]. Nevertheless, several reports demonstrate acetylation and stabilization of p53 in various tumor models associated with the induction of cell cycle arrest and apoptosis [107,108,112]. Consistently, the combined inhibition of HDACs by TSA, and SIRT1 by nicotineamide, reversed the inhibitory effects of cAMP on DNA damage-induced p53 stabilization and apoptosis, providing evidence that p53 acetylation is involved in anti-apoptotic activities [283]. Also, TSA-mediated induction of apoptosis by PUMA upregulation in gastric cancer cells could be achieved, either by direct p53 modulation, or by HDAC3 inhibition, promoting the interaction of the PUMA promoter with p53 [113]. Furthermore, experiments of isogenic HCT-116 colon cancer cell lines with different mutant or wild-type p53 variants demonstrated either p53-dependent or independent antitumor responses depending on the type of HDACi treatment [284,285]. In addition, a backup mechanism of HDACi-induced stabilization of the transcription factor RUNX3 could be demonstrated that induces cell cycle arrest and apoptosis, due to concomitant upregulation of p21 and BID in tumor cells [286,287,288]. Consequently, HDACi may activate p53, but do not unconditionally need p53 for exerting anticancer effects, and p53-dependent as well as independent pathways may contribute to HDACi-mediated apoptotic processes.

In addition to canonical cell death, programmed activation of autophagy can occur alone or in combination in tumor cells expanding the repertoire of HDACi-elicited anti-cancer mechanisms [13,187,202,205,206,207,216,226,289,290]. Recent evidence implies that also p53 is involved in HDACi-inflicted autophagic cell death reflecting the importance of acetylation in autophagy control. Particularly, forapoptosis resistance in p53-deficient malignant cells, HDACi-elicited autophagy could provide an alternative pathway to sustain programmed cell death. The results of our own research studies performed in endometrial sarcoma (ESS) cells suggest that p53 is a major regulatory switch between HDACi-triggered autophagic and apoptotic cell death [13]. In this model, we strived to unearth the molecular cause for the previously published prevalent cell death due to dose-dependent induction of autophagy in ESS-1 cells, in contrast to predominant activation of apoptosis in MES-SA cells, following the treatment with the potent small-molecule pan-inhibitor SAHA [291]. Caspase-dependent and independent cell death resulted thereby in the elimination of 48% MES-SA cells and 80% ESS-1 cells after 24 hours of HDACi administration, respectively, and included prominent p21 upregulation and cell cycle arrest at the G1/S transition phase in both cell types. Consistent with the induction of the autophagic pathway, we were also able to detect a decrease in mTOR expression in the ESS-1 cell line that was not detectable in MES-SA cells [13]. Therefore, we were keen to identify differences in upstream signaling pathways of both cell lines that link mTOR with SAHA-induced autophagy. Upon investigating major autophagic and apoptotic regulators upstream of mTOR, we determined a complete absence of p53 protein coupled with decreased levels of PUMA expression in ESS-1 cells. p53-deficient expression, promoted by a presumable degradation of the p53 transcript, was obviously caused by a novel nonsense mutation (p53^R213X^) that we uncovered in the transactivating domain of p53 and was not present in MES-SA cells. Transfection of ESS-1 cells with wild-type p53 mRNA therefore re-induced apoptosis, as documented by upregulated PUMA, caspases-9, as well as effector caspase-3 and -7 expression, in addition to PARP-1 cleavage. At the same time, expression levels of mTOR were re-elevated accompanied by the return to basic autophagy, as supported by LC3 staining. This HDACi-mediated shift between predominant induction of autophagy or apoptosis was therefore depending on the presence of functional p53 protein. This finding could be further confirmed by repeating the p53-mediated rescue in several other p53-deficient tumor cell lines (such as PANC-1, Jurkat, HL-60, and U937) that previously have been reported to undergo SAHA-induced apoptosis or autophagy.

Similar to our study, HDAC-induced autophagy involving a major regulatory role of the transcription factor p53 was noted in several other reports [281,292,293,294]. Concomitant induction of apoptosis and autophagy occurred in the valproic acid (VPA) and TSA treated p53-deficient pancreatic cancer cells [281]. Intriguingly, the identified mechanism included the ability to reactivate wild-type p53 expression from the non-mutant allele and ERK-mediated stabilization of the oncogenic protein c-Myc; the second previously published effect, which activates cell proliferation in cancer cells, is based on the inhibition of ERK phosphorylation and associated c-Myc expression potentially involving acetylation. Attenuated activation of autophagy evoked by re-established p53 wild-type expression correlated with increased expression of the p53 transcriptionally regulated proteins, p21 and PUMA. Additionally, by screening TSA-treated pancreatic cells comparatively low down-regulation of the anti-apoptotic protein Mcl-1 and reduced ROS production occurred in Panc-1 cells. The Sirt1 and 2-specific deacetylase inhibitor sirtinol has also been attested to effect HDACi-induced cell fate in several studies as it interferes with p53-specific deacetylation. Thus, p53 was implicated in regulating the counterbalance between sirtinol-induced apoptosis and autophagy in MCF-7 breast cancer cells [292]. While sirtinol administration favored increased autophagy as demonstrated by LC3-II expression, increased cytochrome C-mediated apoptotic cell death and cell cycle arrest were provoked by application of the autophagy inhibitor 3-methyladenine resulting in elevated BAX levels, but decreased BCL-2 protein expression. A second study also included the novel Sirt1, -2, and -3 protein inhibitor, MHY2256, in addition to sirtinol inhibition in MCF-7 cells, which resulted similarly in cell death type-I and -II including cell cycle arrest [293]. Here, specifically SIRT1/2-stimulated acetylation of p53 at lysine 382 suppressing MDM2-mediated ubiquitination of p53 was found to result in stabilization and increased functional activity of p53. Similarly, MHY2256 treatment caused MDM2-mediated degradation of p53 in endometrial cancer-derived Ishikawa cells and thereby triggered induction of autophagy as well as apoptosis [294]. MHY2256-elicited apoptosis was associated with elevated BAX and slightly increased BCL-2 expression, cytochrome C release, and elevated PARP-1 cleavage, as well as p21 upregulation that was linked to heightened autophagy in Ishikawa cells. The specific involvement of SIRT1 in the regulation of autophagy has also been confirmed by rescue experiments of homozygous *Sirt1*-deficient mouse embryonic fibroblasts [238]. Only cells that possessed the *Sirt1* wild-type gene allowed re-induction of autophagy under starvation conditions and was potentially promoted by the Sirt1 target proteins ATG5, ATG7, and ATG8 whose acetylation was significantly increased in *Sirt1*-deficient fibroblasts.

The role of p53 as a central factor in coordinating the mode of HDACi-induced cell death is in agreement with a previously determined dual role of p53 in autophagy regulation, depending on its subcellular location in non-transformed cells [295]. In contrast to the nuclear function of p53 in the activation of autophagy, in response to stress conditions, a kind of steady-state function of p53 in arresting the induction of autophagy in the cytoplasm under normal physiological settings was unraveled. Although this novel discovered function of p53 does evidently not involve transactivation-dependent activity but rather direct interaction of the protein with target proteins, it employs also the canonical AMPK–mTOR signaling pathway. In contrast to the nuclear pathway however, inhibition of the AMP-dependent kinase—resulting in activation of mTOR signaling and associated hyperphosphorylation of AMPK, TSC2, acetyl CoA carboxylase, and the mTOR substrate p70S6K—is enforced by cytoplasmic p53 protein. Presently, the involved mechanism is still unclear. Experimental evidence involving mutational analyses indicate that p53 directly interacts with the autophagy-related protein FIP200 (focal adhesion kinase interacting protein of 200 kDa; ATG17) [296]. This interaction inhibits the activation of the ATG13–ULK–FIP200 complex that is responsible for phagopore/autophagosome formation and is indispensable for nutrient starvation-induced autophagy. Interestingly, p53 variants with a loss-of-function promoted the activation of autophagy, while several variants with gain-of-function mutations could still fulfill cytoplasm-controlled inhibition of autophagy. As a proof-of-concept, the drug-induced inhibition of basal cytoplasmic p53, or conversely, a nuclear export domain-deficient form of p53 either exerted an activating or inhibiting influence on autophagy, respectively. Confirmation of this mechanism comes from embryonal carcinoma cells where genetic deletion of p53 activated autophagy involving Beclin-1-mediated degradation of p53 via USP10 and USP13 ubiquitin-specific peptidases in a feedback loop [297]. Evolutionary, cytoplasm-induced transcription-independent autophagy seems to represent a backup mechanism, in case p53 is mutated, to provide increased resilience towards metabolic stress and ensure cell survival.

### 6.3. Balanced Control of HDACi-Mediated Apoptosis and Autophagy by p53

Programmed cell death by apoptosis and autophagy are in general two different stress-induced biological responses that are mutually exclusive and commonly block each other as they are governed by two distinct genetic programs. Nonetheless, in order to avoid concurrent activation these processes have to undergo coordinated regulation. Thus, initiation of the apoptotic program, as one of the most frequently ensued cell death modalities, also involves the activation of a series of caspases. By proteolytic cleavage of several key regulatory proteins of the autophagic pathway, caspases are likewise able to contribute to the shutdown of autophagy. Thereby, the cytoprotective response of autophagy might be suppressed whereas apoptotic cell death is enhanced. Anti-apoptotic mechanisms, conducted by the autophagic program include the selective degradation of ubiquitinylated cytoplasmic pro-apoptotic proteins. Alternate effects involve targeting of specific molecules such as SQSTM1/p62 that stimulate ROS generation and ROS-induced apoptosis when present in excess amounts in the cell [298,299]. The ubiquitin-binding scaffold protein SQSTM1/p62, is an autophagosome cargo protein that targets other interacting proteins for selective autophagy. It binds to LC3 and thereby acts as a signaling hub that controls cell survival, apoptosis, and inhibition of tumorigenesis by autophagy [300,301,302,303]. Furthermore, the removal of mitochondria by mitophagy can lower the propensity of the cell for intrinsic apoptosis [184]. Under certain conditions which are not well elaborated however, combined induction of autophagic and apoptotic cell death has been observed which is also relevant for many studies involving HDACi-treated cancer cells.

On the molecular level, this dual activation of autophagy and apoptosis involves modifying pro-apoptotic members of the BH3-only family that compensate anti-apoptotic BCL-2 family proteins. These proteins directly interact with the BH3 domain of BECLIN-1 and block BECLIN-1–dependent autophagic activation [304,305]. Either transcriptional downregulation of BCL-2, BCL-XL, and MCL-1, or transcriptional upregulation of BAX, BAD, BNIP3, or PUMA, releases BECLIN-1 from its protein complex that initiates autophagy [306]. Consistently, p53-regulated pro-apoptotic proteins have also been identified as autophagy-promoting proteins [307,308]. As a debated mechanism of autophagy activation, p53 was described to stimulate the phosphorylation of BCL-2 [309]. This causes dissociation of the BECLIN-1/BCL-2 complex since BCL-2 functions as an anti-apoptotic as well as an anti-autophagic protein via its inhibitory interaction with BECLIN-1. The released BECLIN-1 (ATG6) then stimulates phagophore, and finally autophagosome formation, by recruiting and interacting with many proteins [310]. Contrastingly, there is evidence suggesting that the effect of autophagy-promoting BCL-2 family members is owned to their antagonizing inhibitory activity on the apoptosis mediators BAX and BAK. Thus, when BAX and BAK are absent in the cell, BCL-2 family members have no measurable impact on autophagy [311]. A second path is mediated by the upregulation of DRAM (damage-regulated autophagy modulator) through stress-activated p53 [312]. As a lysosomal protein regulating autophagosome-lysosome fusion and direct mediator of autophagy and apoptosis, DRAM can interfere with several different stages of autolysosome formation [313].

Recent reports indicated that also the frequently cancer-deregulated MAPK members, JNK and p38, have a master regulatory role in these processes [309]. MAPK members regulate a variety of cellular activities and can modulate processes, such as proliferation, differentiation, apoptosis and autophagy in response to extracellular stimuli that include UV irradiation, inflammatory cytokines, protein synthesis inhibitors, DNA damaging agents, or chemotherapeutic agents. Interestingly, both of the above specified cell death crosstalk mechanisms were promoted through the activation of p53 and JNK pathways in 2-methoxyestradiol-treated Ewing sarcoma cells [314]. Thus, p53 was assumed to regulate JNK activation which itself regulates DRAMin its function as a pro-apoptotic and pro-autophagic protein. The eminent role of DRAM in 2-methoxyestradiol -mediated autophagy and apoptosis could be demonstrated by silencing of DRAM which suppressed the induction of both processes. Additionally, BCL-2 phosphorylation resulting in the dissociation of the BECLIN-1-BCL-2 complex could be confirmed in this study. As a further rare p53-independent mechanism, calpain-mediated ATG5 fragmentation was inducing mitochondria- and caspase-dependent apoptotic cell death, thereby representing a molecular link between autophagy and apoptosis [315]. Interacting apoptosis and autophagy, including involvement of ATG proteins through caspase- and calpain-1-induced cleavage, were reported, for example, in a case of MGCD0103-treated primary chronic leukocytic leukemia cells, where the PI3K-AKT-mTOR pathway was also activated [208]. The mocetinostat-mediated inhibition of autophagy also involved direct transcriptional modulation of the expression of critical autophagy genes.

As already specified in Section 5.1, for the enhancement of concurrent stimulation of apoptosis and autophagy, also HDACi prevalently impede the equilibrium between pro- and anti-apoptotic proteins, and have been documented to facilitate the down-regulation of anti-apoptotic genes (such as *BCL-2*, *BCL-XL*, *XIAP*, *MCL-1*, *Survivin*) [112,113,119,135,157,158]. BCL-2/BECLIN-1-dependent autophagic cell death has not been thoroughly investigated in HDACi-treated cancer cells but is nevertheless presumed to mediate combined activation of both cell types in most cases. Thus, as we previously reviewed, Bcl-2 downregulation was indicated in several reports involving HDACi-mediated dual activation of apoptosis and autophagy involving either p53-specific acetylation or other/unknown mechanisms [15,292,293,316]; On a similar note, also Beclin-1 upregulation has been determined in a few cases among these summarized studies [15,203,204,317]. A direct support for this mechanism comes from a study applying a knockdown of anti-apoptotic mRNAs for *BCL-2*, *BCL-XL*, and *MCL-1* or using a BCL-2 family inhibitor (GX15-070) in pancreatic cancer cells with combined administration of SAHA or sodium valproate and sorafenib; these inhibitory agents induced autophagy and intrinsic apoptosis, thus bypassing extrinsic apoptosis resistance [318]. In addition, the coupling of apoptosis to suppressive autophagy has been proven by combined with the HDACi-mediated upregulation of BIM and BH3 mimetic-induced liberation of BIM from anti-apoptotic proteins, a process that is normally reliant on p53 [319,320].

Further studies support ROS-induced dual activation of apoptosis and autophagy including a report where autophagy was downregulated [203,219,321]. With respect to MAPK-activated cell death by HDACi, a key regulatory role for ROS-induced p38 MAPK in the balance between apoptosis and autophagy in MS-275 treated HCT116 colon cancer cells could be clearly demonstrated [322]. The type of induced cell death correlated with the duration of HDACi treatment and the resulting expression levels of p38 in this study. While short-term treatment and low ROS-induced ERK activation via p38 resulted in ATG7-induced autophagy, the apoptotic pathway could only be induced by high levels of phosphorylated ERK, JNK, and AKT proteins, provoked by long-term treatment of more than 48 hours or by a knockdown of ATG7. As already detailed above in Section 5.2, the Nrf2-miR-129-3p-mTOR Axis was described to regulate HDACi-induced autophagy [218]. As generally the Nrf2 signaling pathway is activated according to to the MAPK, JNK and p38, a link to the canonical mTOR-regulated pathway of autophagy was established by this report and might also connect to p53. This may be of high relevance as the regulation of Nrf2-mediated autophagy is one mechanism of HDACi-caused chemoresistance.

Nevertheless, beside p53 and MAPK other central coordinators that balance HDACi-induced cell death, favoring either one of both types of cell death, or concurrent activation of apoptosis and autophagy, are very likely to exist. Accordingly, p53-independent induction of p21 expression in combination with dual activation of apoptosis and autophagy have been exemplified in apicidin-treated YD-8 and YD-10B human oral squamous carcinoma cells (OSCC) without defining its molecular causes [282]. Many studies that report simultaneous activation of both types of HDACi-induced cell death also evidenced increased acetylation of the core histones H3 and H4 (for review see [15]). However, as these have been associated with increased transcription of distinct genes implicated in tumor growth suppression it is not clear whether these have a role as HDACi-mediating cell death regulators [81,323]. Therefore, the question of which factors and which exact molecular mechanisms influence the cell’s decision to modulate the interplay and balance between the different modes of HDACi-elicited cell death awaits further detailed research.

## 7. Perspectives and Future Directions

Identifying the mechanisms of programmed cell death is one of the key requirements to achieve progress, not only in unraveling pathogenetic insights, but also in the development of novel therapeutic strategies for diseases such as cancer. Traditionally, in the last few decades cancer therapy has mainly focused on the enhancement of cellular apoptosis. More recently, clinical strategies for cancer therapy have enforced HDACi administration combined with inhibitors of autophagy. The ability to sensitize apoptosis-resistant tumor cells by the disruption of autophagy was considered a promising route for cancer therapy as this process augments the pro-apoptotic effects of HDACi. In addition, to restrain the extents of tumor necrosis and inflammation, autophagy might be required for the cancer cell to deal with metabolic stress and cytotoxicity during chemotherapy. Furthermore, by expediting the autophagic pathway in advanced stages of the cancer cell, autophagy may promote cell death by mostly non-elucidated mechanisms. This might be also a significant preference for apoptosis resistant cancer cells due to the inactivated caspase-mediated pathways or specifically acquired HDACi-resistance. In this context, the disruption of the autophagic program will facilitate tumor survival. In line with this, it is of crucial importance to define the factors and mechanisms that influence the balance between HDACi-elicited apoptosis, autophagy or even necrosis in the cancer cell. Studying the detailed mechanisms of the modulation of autophagy and apoptosis may lead to a better understanding of the discriminatory role of different p53 variants in controlling cell death and provide the potential to bypass the resistance of cancer cells.
